# Assessing equity in health, wealth, and civic engagement: a nationally representative survey, United States, 2020

**DOI:** 10.1186/s12939-021-01609-w

**Published:** 2022-01-28

**Authors:** Thomas J. Stopka, Wenhui Feng, Laura Corlin, Erin King, Jayanthi Mistry, Wendy Mansfield, Ying Wang, Peter Levine, Jennifer D. Allen

**Affiliations:** 1 Public Health and Community Medicine, Tufts University School of Medicine, 136 Harrison Ave, Boston, MA 02111 USA; 2 Clinical and Translational Sciences Institute, Tufts University School of Medicine, 35 Kneeland St, Boston, MA 02111 USA; 3 Department of Community Health, Tufts University, 574 Boston Ave, Medford, MA 02115 USA; 4 Department of Civil and Environmental Engineering, Tufts University School of Engineering, 200 College Ave, Medford, MA USA; 5 Tisch College of Civic Life, Tufts University, Medford, MA USA; 6 Cummings School of Veterinary Medicine, Tufts University, 200 Westborough Rd, North Grafton, Medford, MA 01536 USA; 7 Eliot-Pearson Department of Child Study & Human Development, Tufts University, 105 College Ave, Medford, MA USA; 8 Ipsos, 2020 K Street, NW, Suite 410, Washington, DC, 20006 USA

**Keywords:** Equity, Health, Wealth, Civic engagement, Nationally representative survey

## Abstract

**Background:**

The principle of equity is fundamental to many current debates about social issues and plays an important role in community and individual health. Traditional research has focused on singular dimensions of equity (e.g., wealth), and often lacks a comprehensive perspective. The goal of this study was to assess relationships among three domains of equity, health, wealth, and civic engagement, in a nationally representative sample of U.S. residents.

**Methods:**

We developed a conceptual framework to guide our inquiry of equity across health, wealth, and civic engagement constructs to generate a broad but nuanced understanding of equity. Through Ipsos’ KnowledgePanel service, we conducted a cross-sectional, online survey between May 29–June 20, 2020 designed to be representative of the adult U.S. population. Based on our conceptual framework, we assessed the population-weighted prevalence of health outcomes and behaviors, as well as measures of wealth and civic engagement. We linked individual-level data with population-level environmental and social context variables. Using structural equation modeling, we developed latent constructs for wealth and civic engagement, to assess associations with a measured health variable.

**Results:**

We found that the distribution of sociodemographic, health, and wealth measures in our sample (*n* = 1267) were comparable to those from other national surveys. Our quantitative illustration of the relationships among the domains of health, wealth, and civic engagement provided support for the interrelationships of constructs within our conceptual model. Latent constructs for wealth and civic engagement were significantly correlated (*p* = 0.013), and both constructs were used to predict self-reported health. Beta coefficients for all indicators of health, wealth, and civic engagement had the expected direction (positive or negative associations).

**Conclusion:**

Through development and assessment of our comprehensive equity framework, we found significant associations among key equity domains. Our conceptual framework and results can serve as a guide for future equity research, encouraging a more thorough assessment of equity.

**Supplementary Information:**

The online version contains supplementary material available at 10.1186/s12939-021-01609-w.

## Introduction

Individuals, communities, and societies can experience equitable or inequitable conditions across multiple domains including health, wealth, and civic engagement. Traditional research approaches typically focus on only one or perhaps two of these dimensions at a time. For example, it is well established that income and wealth (i.e., net worth and assets) are directly associated with health outcomes [1–3], and that inequalities in wealth are associated with inequalities in health as assessed by metrics such as life expectancy [4–6]. The associations among these domains can appear bidirectional. For example, adverse mental health damages individuals’ economic circumstances, and poverty contributes to adverse mental health [7].

Similarly, accounting for the health and civic engagement domains, research indicates that increased well-being, physical health and mental health are associated with increased civic engagement [8], and that poor health earlier in life is associated with lower levels of civic engagement later in life [9, 10].

Finally, considering the wealth and civic engagement domains, researchers have consistently found associations between wealth and social class, on one hand, and civic engagement behaviors, on the other [11]. Income inequality has risen as volunteering and associational membership have decreased [12], and social capital and civic engagement are negatively associated with income inequality [13–15].

While the connections between health and wealth, civic engagement and health, and wealth and civic engagement have been explored previously, there is a paucity of research that explores the interrelationships between all three domains, that is between equity in health, wealth, and civic engagement. To help fill this gap in the assessment of holistic connections across all three domains, we conducted a cross-sectional, online survey between May 29–June 20, 2020 designed to be representative of the adult U.S. population. In this paper, we present our guiding conceptional equity framework, our methodological approach, and salient results from our quantitative assessment.

### Conceptual framework

We developed a conceptual framework to guide our interdisciplinary research and inform development of our study. For our framework, we adapted elements from the World Health Organization’s Commission on Social Determinants of Health’s (CSDH) framework [16] and the social ecological model [17, 18]). We adapted the CSDH model following extensive discussion with our interdisciplinary team that includes experts in community health, economics, education, epidemiology, human development, philosophy, One Health, psychology, and statistics [19].

**Fig. 1:**
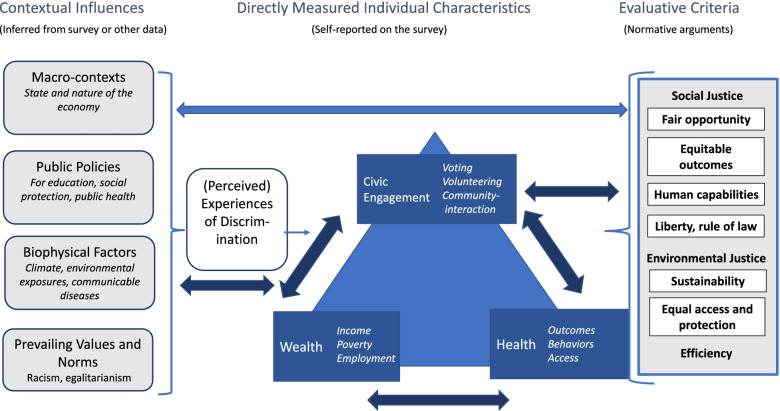
Conceptual Framework: Equity in Health, Wealth, and Civic Engagement

In our final framework (Fig. 1), the central triangle presents major categories of variables that we directly measured in our survey: health, wealth, and civic engagement. The figure also provides examples of specific variables within those categories, such as health outcomes, income, and voting, respectively. The arrows suggest that these three domains are empirically associated, which we hypothesized based on the literature, and set out to test in our data.

On the left-hand side of the panel, the figure depicts contextual factors that may influence individuals within the target population of interest and the relationships among the individual-level variables we measured. For example, the survey was conducted during a pandemic and a recession, when large-scale biomedical and economic conditions likely affected individuals’ responses. The model suggests that people are also affected by policies, at all levels of government, and by other’s treatment of them. These experiences, such as discrimination at the individual, institutional, and structural levels, can mediate associations between contextual factors and individual characteristics. For some of these contextual factors, respondents to our survey serve as informants. For example, they report on whether they experience discrimination, which is suggestive evidence of the degree of the actual discrimination they face. For other variables, we can link external data (e.g., measured environmental conditions or policies passed in a region) to individuals to understand the environmental or political context. Our conceptual framework allows us to investigate various aspects of equity understood as relative positions in a stratified social structure, or concrete experiences of inequity, or both [20]. To explore relative positions, we can examine the variables in the survey that measure conditions that may be distributed unequally in society, such as income, voter turnout, and health outcomes. Such findings provide a basis for discussing whether circumstances are inequitable. To explore concrete experiences of inequity, we can examine data on discrimination, perceived treatment in the healthcare system, and the responsiveness of institutions, among other measures. Statistical models can then investigate the relationships among demographic characteristics (e.g., race/ethnicity, age, and gender); relative positions in the social structure; concrete experiences of inequity; and health, wealth, or civic engagement outcomes.

From our cross-sectional survey data (see Methods and Results below), we can indicate whether particular populations are equal or unequal in various respects. For this purpose, equality is defined as an empirical matter, a mathematical relationship between two variables. In contrast to equality, equity is a normative or value-laden concept, debated by people who hold different normative principles. For example, according to the survey, people who identify as female are 10 percentage points more likely to suffer from depression than those who identify as male. Determining whether a 10 point difference in the prevalence of a specific health condition by gender is a sign of inequity, and why, requires an argument based on normative principles and reasons. To argue that the prevalence of a given health condition should be the same by gender implies a broader theory of equity that needs justification, and it raises further questions: Is any incidence of an adverse health condition acceptable? Would the situation be more equitable if both men and women reported equal, but higher, rates of the condition? Do the reasons for the difference matter when considering whether the outcome is equitable? If the health condition could be cured without solving the causes of it, would that be equitable?

The right-hand panel of the framework presents evaluative criteria that are influential in public debates, although also controversial. For instance, focusing on the fairness of opportunities versus outcomes can yield different judgements. Efficiency refers to the social cost of obtaining a benefit, such as public health, whereas liberty is sometimes understood as a right that must be protected regardless of cost/benefit efficiency.

The model suggests hypotheses about these types of questions, and others, that can be tested through subsequent analyses relating normative arguments to empirical data. In our conceptual framework, the double-headed arrow between the central and right-hand elements of the model represents a dialogue between normative principles and empirical evidence. Such a dialogue is a feature not only of our interdisciplinary research team but also of a healthy public debate. Facts should influence people’s values; and values should influence what is measured and how the data are interpreted [21].

## Methods

### Survey administration

We collaborated with Ipsos, a social science research company, to conduct the survey using the web-enabled KnowledgePanel®, the largest, online, probability-based panel designed to be representative of the U.S. population. Initially, participants were chosen by a random selection of telephone numbers and residential addresses. Persons in selected households were then invited by telephone or by mail to participate in the web-enabled KnowledgePanel. For people who did not already have Internet access but agreed to participate, Ipsos provided a laptop/netbook and Internet Service Provider (ISP) connection at no cost to the participant. People who already had computers and internet service were permitted to participate using their own equipment. Panelists then received unique log-in information for accessing surveys online. They were sent emails a few times each month inviting them to participate in research. Due to the probability-based recruitment methodology Ipsos used, samples selected from the KnowledgePanel were representative of the US population with a measurable level of accuracy—a feature not obtainable from nonprobability or opt-in online panels (for comparisons of results from probability versus nonprobability methods, see [22–24]).

In our final instruments and analyses, we examined measures from both the survey we developed with our interdisciplinary team (the “Tufts equity survey”) and Ipsos’s own annual surveys of the KnowledgePanel (“Ipsos profile surveys”). The latter collect information on personal and household characteristics, personal health, health coverage and attitudes, lifestyle, finance, politics, media usage, and other subjects.

### Equity survey sampling frame

Participants were non-institutionalized adults aged 18 years or older living in the United States. A total of *n* = 1980 KnowledgePanel members were invited to complete the equity survey.

We fielded the equity survey in English and Spanish from May 29 to June 10, 2020. On day three of the field period, an automatic email reminder was sent to all non-responding sample members. Additional email reminders were sent to non-Hispanic Black and Hispanic non-responders on day 11 of the field period in an effort to maximize the survey completion rate from these demographic groups. The median completion time of the equity survey was 17 min. Upon completion, qualified respondents received their standard incentive payment (for most respondents, 1000 points, the cash-equivalent of $1 and an entry into the KnowledgePanel sweepstakes for completing a survey longer than 15 min).

### Response rates

From the random sample of 1980 panel members, 1267 responded to the invitation, and all qualified for the survey, yielding a final stage completion rate of 64.0% and a qualification rate of 100% percent. The panel recruitment rate (for agreeing to join the panel) for this study, reported by Ipsos, was 11.9% and the profile rate (for completing the profile survey collecting key demographics for sampling and weighting, required before panel members can complete any other surveys) was 61.1%, resulting in a cumulative response rate (recruitment rate x profile rate x survey completion rate) of 4.7%.

### Survey development

Survey items focused on the domains of equity in health, wealth, and civic engagement were developed through a collaborative process within our interdisciplinary team. Broadly, our operational definition of equity in these three domains includes a holistic understanding of equity, including overall health measures, specific chronic and communicable disease outcomes, income, education, home ownership and poverty measures, and active and passive service and civic activities (e.g., volunteer work, voting, collaboration to solve a community problem, protesting) in local communities. Where possible, standardized survey items were employed. Final survey items were programmed, piloted, and fielded to our target sample.

The variables were heterogeneous in type. Some measured psychological states, such as the sense that one suffers discrimination. Psychological states are often constructs that are best measured with multiple survey items that form meaningful scales. In contrast, some variables measured objective assets, such the size of one’s annual income and whether one owns a home. Such assets are additive—a greater total implies more wealth—but they may not scale because they are not psychological constructs. In fact, a person could hold multiple assets and yet not feel wealthy. Finally, some variables were concrete behaviors, such as voting in the 2000 election. Voting is an example of a way of influencing institutions--and so is protest. Like various forms of wealth, voting and protest are additive: voting in multiple elections plus protesting add up to a higher level of engagement. However, whether responses to survey questions about such forms of participation form scales is not our focus, because we are interested in actual engagement rather than the psychological construct of feeling civically engaged. Our overall strategy is not to investigate or report scales, because only a few of our variables measure psychological constructs. Instead, we aggregate the variables using a structural equation model applied to the population as a whole, as described below.

### Measures

#### Health measures

We assessed self-rated general health, clinician-diagnosed history of specific infectious diseases (e.g., “Have you ever been told by a health care professional that you had COVID-19**,** the human immunodeficiency virus [HIV], the hepatitis C virus [HCV]), chronic conditions (chronic kidney disease, chronic obstructive pulmonary disease [COPD] or asthma, heart conditions [heart attack, heart disease, or other heart condition], pulmonary arterial hypertension, high blood pressure, diabetes or pre-diabetes, and non-alcoholic fatty liver disease), and history of mental health conditions (anxiety, depression, mood disorder, schizophrenia).” Participants also reported their height and weight, from which we calculated individual body mass index (BMI) and obesity status (BMI ≥ 30 kg/m^2^). We included behavioral health indicators: whether the respondents smoked ≥100 cigarettes in their lifetime, whether they ever vaped, and whether they have been told by healthcare professionals that they have alcohol use disorder, substance use disorder, or opioid use disorder. At the time of survey development, there were few validated items to assess COVID-19 experiences. We included questions from the standardized CoRonavIruS Health Impact Survey (CRISIS) [25] to assess whether a participant or a family member had been tested (e.g., “Have you ever received a test for the Coronavirus?”) and/or diagnosed with COVID (i.e., “Have you personally been told by a healthcare professional that you were infected with Coronavirus?”). Response options were “yes”, “no”, “don’t know”. We also included items to assess COVID-related behaviors (e.g., social distancing [“Have you tried to isolate yourself from contact with other people because of Coronavirus?”], vaccine intentions [“If a vaccine became available to prevent the Coronavirus, would you get it”]) and the same response options were provided.

#### Wealth measures

Our wealth outcomes consisted of educational attainment (less than high school, high school, some college, Bachelor’s degree or higher), home ownership status (owned or being bought by you or someone in your household, rented for cash, occupied without payment of cash rent), primary source of health insurance (employer sponsored insurance, Medicare/Medicaid, Health Insurance Marketplace, Veteran’s Affairs, or other health insurance), and annual household income (<$10,000, $10,000–$14,999, $15,000–$24,999, $25,000–$34,999, $35,000–$49,999, $50,000–$74,999, $75,000–$99,999, $100,000–$149,999, $150,000–$199,999, ≥$200,000), employment status (working, laid off/looking, retired/disabled/other not working). We took each income interval’s median value to convert the income information into a percentage of the 2020 federal poverty level (FPL) accounting for family size [26].

#### Civic engagement measures

We define civic engagement as all the ways that people act to maintain or improve their communities and political regimes. It encompasses horizontal relationships among members of communities and vertical relationships between residents and institutions, e.g., voters casting ballots to choose leaders. It has behavioral components, such as voting, volunteering, and expressing political opinions, as well as affective components, such as a sense of efficacy—both personal and collective—or the confidence to make change ([27, 28].)

The survey included measures of voting, news consumption, opinions, financial donations, and participation in specific groups and movements, including political parties, unions, and a list of 25 named activist organizations. We also included three concrete behavioral measures: canvassing, serving as a nonprofit board member, and planning to vote in the 2020 election (using the item that Gallup uses to assess likelihood of voting on a 10-point scale)[29]. We also added three general civic engagement items:

1. Have you ever worked together informally with someone or some group to solve a problem in the community where you live? [30] (Community problem-solving). [Response options: Yes, within past 12 months; Yes, but not within past 12 months, No, Refused.]
2. Please think about the problems you see in your community. How much difference do you believe YOU can personally make in working to solve the problems you see? (Personal efficacy). [Response options: Refused; No difference at all; Some difference; A great deal of difference].
3. How much difference do you believe you and other members of your community can make if you work together? (Collective efficacy). [Response options: Refused; No difference at all; Some difference; A great deal of difference].

We created a composite measure for civic engagement that included activities tied to activism or community organizing (e.g., attended a political protest or rally; contacted a government official; served on a committee for a civic, non-profit or community organization; commented about politics on a message board or Internet site; held a publicly elected office; shared opinion about a town or community issue at a public meeting; signed a petition; volunteered or worked for a presidential campaign; volunteered or worked for a political candidate other than a presidential campaign; volunteered or worked for a political party, issue, or cause; or written a letter or email to a newspaper/magazine or called a live radio or TV show).

#### Perceived discrimination measures

We included seven close-ended survey items to measure perceived discrimination and one open-ended. The close-ended items were adapted from the Perceived Discrimination Scale developed by Williams, Neighbors, and Jackson, [31], for the 1995 Detroit Area Study, which collected face-to-face interviews with 1139 adults residing in three counties of Michigan. The original two-part scale was used to measure (a) major experiences of unfair treatment, as well as (b) more chronic, routine, and relatively minor experiences of unfair treatment. The Perceived Discrimination Scale [31] has been validated in adult samples across multiple studies, with the internal reliability of the Everyday Discrimination subscale ranging from 0.80 to 0.90. The convergent validity for the scale has been established with other scales of perceived stress, depression, and negative affect and social strain diary data in a sample of older adults in the Pittsburgh metro area [32], and to assess a self-report measure for population health research on racism and health tied to discrimination [33]. In addition, this scale is one of the resources recommended by the CDC in its 2007 publication “Expanding our understanding of the psychosocial work environment: a compendium of measures of discrimination”.

Of the seven close-ended perceived discrimination survey items included in the Tufts equity survey, four items represent major discrimination experiences, one of which is from the original Perceived Discrimination Scale, and three items, all from the original scale, represent everyday experiences of discrimination. The first question asks: “In your day-to-day life, how often have any of the following things happened to you?**”** with prompts such as “You are treated with less courtesy or respect than other people are”, and “You are threatened or harassed.” Respondents are asked to estimate the frequency of each of these experiences for Major Perceived Discrimination on a 4-point scale (1 = never, 2 = once or twice, 3 = 3 times or more times, or 4 = Not this year, but in the past). Respondents answered the frequency questions for Everyday Perceived Discrimination on a 4-point scale (1 = Never, 2 = Rarely, 3 = Sometimes, 4 = Frequently). In the current study, we assessed frequencies and percentages for response options for the seven items.

After each of the items, respondents are asked to select the perceived reason for the discrimination from a set of six options (your race/ethnicity, your gender, your religion, your health, your sexual orientation, your economic situation).

### Linkage to other data

Our data captured individuals’ current and childhood exposure to community-level economic, social, and environmental factors at the ZIP Code and county-level. Data were linked to participants based on their current ZIP Code and county of residence and their self-reported ZIP Code and county at 10 years of age. We obtained data for economic and social community-level factors (e.g., percentage of a ZIP Code with a given race/ethnicity, percentage of a ZIP Code with different levels of educational attainment, median household income for the ZIP Code, and residential racial segregation) from the U.S. Census Bureau’s American Community Survey (United States Census Bureau, 2015–2019). We also obtained data on social capital (e.g., family unity, family interaction, social support, community health, institutional health, collective efficacy, and philanthropic health) at the state and county level [34]. The dataset included standard state and county level indices for these factors, as well as data on the factors contributing to each indicator and state and county-level socioeconomic and health benchmarks (e.g., incarceration rates, relative and absolute immobility, unemployment rate, percent with housing costs > 35% of income, percent of children receiving public assistance, on-time graduation rate, percent diabetic, and percent who smoke).

We obtained environmental data from multiple sources: Air pollution data (particulate matter < 2.5 μm [PM_2.5_] in aerodynamic diameter and nitrogen dioxide) were available at the census tract level. These modeled exposure estimates were derived from annual average PM_2.5_ models developed for the global burden of disease effort. We used the North-American-specific models developed at 0.01 × 0.01-degree resolution to calculate the census tract average PM_2.5_ [35]. We obtained temperature data (daily average, minimum, maximum; 4 km × 4 km resolution) from the PRISM Climate group at Oregon State University [36, 37]. Heatwave days were calculated on a per-pixel basis as the number of days during the warm season (May–September) in which the maximum temperature exceeded the pixel-specific 95th percentile for the warm season maximum temperature for 1999–2018 for two or more consecutive days. Pixel values were averaged across tracts and counties and rounded to the nearest whole number to get the number of heatwave days per county. Proximity to greenspace and access to open space were derived using the normalized difference vegetation index (NDVI; an index that indicates photosynthetic activity in plants). We used the 16-day NDVI composites from the Moderate Resolution Imaging Spectroradiometer (MODIS) sensor at 250 m resolution onboard the Terra satellite (MOD13Q1; mean county-level annual maximum NDVI) [38]. Toxic waste site data were available from the Environmental Protection Agency (EPA) Toxic Release Inventory website [39]. We calculated the number of toxic waste sites per county.

### Sample weighting

In order to ensure a balanced representation of survey participants from across the U.S., we incorporated sample weighting techniques in advance of recruitment. KnowledgePanel members represent the U.S. adult population with respect not only to a broad set of geodemographic indicators, but also for hard-to-reach adults (such as those without internet access or Spanish-language-dominant Hispanics) who are recruited in representative proportions. Consequently, the raw distribution of KnowledgePanel mirrors that of the U.S. adults fairly closely, barring occasional disparities that may emerge for certain subgroups due to differential attrition.

To select the general population sample for this study, Ipsos used its patented methodology developed to ensure that all samples behave as an equal probability of selection method samples. Briefly, this methodology started by weighting the pool of active members to the geodemographic benchmarks secured from the March 2019 supplement of the U.S. Census Bureau’s Current Population Survey along the geodemographic dimensions listed below [40]. Using the resulting weights as measures of size, a probability-proportional-to-size (PPS) procedure was used to select the study sample. It is the application of this PPS methodology with the imposed size measures that produced the fully self-weighting sample, for which each sample member carried a design weight of unity.

The geodemographic dimensions used to weight the active panel members for computation of size measures include: gender (female, male); age (18–29, 30–44, 45–59, ≥60 years); race/ethnicity (Hispanic, Non-Hispanic Black, Non-Hispanic White, Non-Hispanic Other, 2+ Non-Hispanic races); educational attainment (less than high school, high school, some college, Bachelor’s and beyond); census region (Northeast, Midwest, South, West) [41]; annual household income (under $10 k, $10 K to <$25 k, $25 K to <$50 k, $50 K to <$75 k, $75 K to <$100 k, $100 K to <$150 k, and ≥ $150 K); home ownership status (own, rent/other); metropolitan area (yes, no) [40]; and Hispanic origin (Cuban, Mexican, Puerto Rican, other, non-Hispanic).

### Study-specific post-stratification weights

Once all survey data were collected and processed, design weights were adjusted to account for differential nonresponse. Using geodemographic distributions obtained from the U.S. Census Bureau's Current Population Survey and American Community Survey, we applied an iterative proportional fitting (raking) procedure to produce the final weights [40, 42]. We used the following benchmark distributions of U.S. adults age 18 and older from the most recent Current Population Survey March Supplement (2019) for the ranking adjustment of weights, and we used the 2018 ACS language proficiency benchmarks to adjust weights for Hispanic respondents: gender (female, male) by age (18–29, 30–44, 45–59, ≥60 years); race/ethnicity (Non-Hispanic White, Non-Hispanic Black, Non-Hispanic Other, Hispanic, 2+ Non-Hispanic races); census region (Northeast, Midwest, South, West) by metropolitan status (metro, non-metro); education (less than high school, high school, some college, Bachelor’s or higher); annual household income (under $10 k, $10 K to <$25 k, $25 K to <$50 k, $50 K to <$75 k, $75 K to <$100 k, $100 K to <$150 k, and ≥ $150 K); and language proficiency (Non-Hispanic, English proficient Hispanic, bilingual Hispanic, Spanish proficient Hispanic).

In the final step, we examined calculated weights to identify outliers at the extreme upper and lower tails of the weight distribution, and we determined no trimming of outliers was needed. The resulting weights were then scaled to aggregate to the total sample size of all eligible respondents. The design effect was 1.2487.

### Statistical analyses

**Fig. 2:**
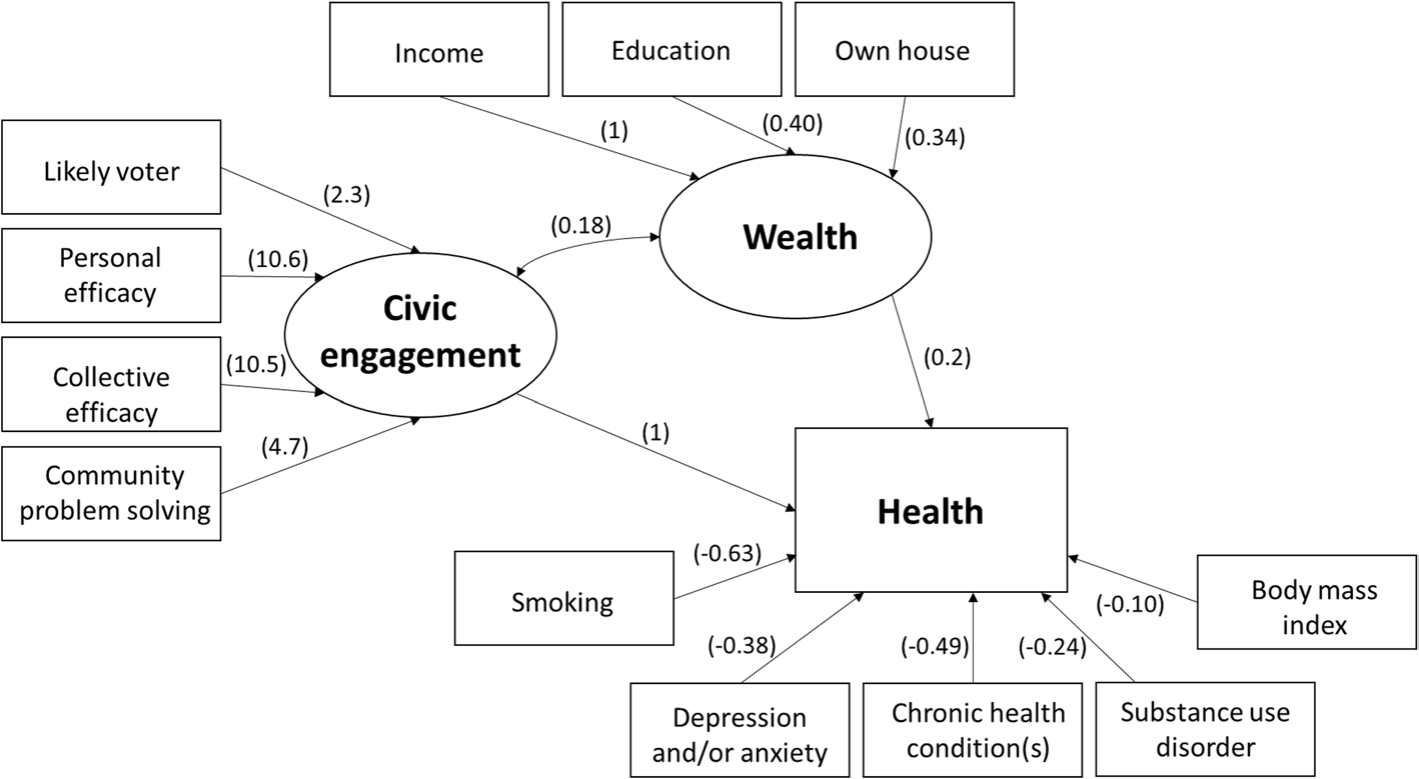
Structural equation model relating health, wealth, and civic engagement. Values in parentheses represent beta coefficients. Ovals represent latent constructs and rectangles represent measured variables. Values = 1 were constrained

First, to understand the characteristics of the study sample, we assessed measures of central tendency and frequency distributions of specific measures of health, wealth, and civic engagement. Second, to verify the representativeness of our sample, we compared distributions of demographic, wealth, and health characteristics of our sample to those from the 2020 Current Population Survey [43]. In these descriptive analyses, we retained answers of “don’t know” or “refused” as separate categories (not treated as missing). Third, we used a structural equation model (SEM) to empirically assess the relationships among health, wealth and civic engagement constructs proposed in Fig. 1. The purpose of using SEMs is to empirically test theoretical relationships among measured variables that are thought to represent related latent constructs [44]. In our illustrative example of a structural model (Fig. 2), we represented the health construct empirically using the self-reported physical health variables (Table 1, shows all levels of the variables used for the SEM analysis). The health construct was a function of five other health variables: (1) body mass index; (2) smoked > 100 cigarettes ever; (3) diagnosed depression or anxiety; (4) substance use concern; and (5) self-reported diagnosis by a healthcare professional of chronic disease. Additionally, the health construct was a function of two latent variables, wealth and civic engagement, that were allowed to have a partial correlation with each other. The wealth latent variable was defined by three indicators: (1) income; (2) educational attainment and (3) home ownership (Table 1). The civic engagement construct was defined by four indicators: (1) self-reported likely voter; (2) self-reported personal efficacy to solve problems in community; (3) self-reported collective efficacy to solve problems in community; and (4) self-reported informal collaboration to solve a community problem (Table 1). The SEM was run using *gsem* in Stata v.16 with robust standard errors and survey weighting. We used a logit link function with a Bernoulli distribution for all dichotomous variables and a logit link function with an ordinal distribution for all categorical variables with ≥3 categories. We also generated a correlation matrix of coefficients within the SEM and we estimated separate logistic regression models to examine bivariate associations between each indicator variable (i.e., the measured covariates for health, wealth, and civic engagement from Table 1) and the self-reported physical health outcome variable.

**Table 1:** Variable definitions for the structural equation model

Construct	Measured variable	Levels (unweighted n)
Health (measured)	Self-reported physical health	very good or excellent (635) poor, fair, or good (621)
Measured covariates for health	Body mass index from self-reported height and weight	continuous; mean = 28.3
	Ever smoked > 100 cigarettes	yes (478) no (786)
	Diagnosed depression or anxiety	yes (211) no (1017)
	Self-reported being told by a healthcare professional that they had any of: a substance or alcohol *or* opioid-use disorder *or* self-reported using prescription pain medication not prescribed to them by a doctor	yes (97) no (1109)
	Self-reported diagnosis by a healthcare professional for any of: chronic kidney disease, chronic obstructive pulmonary disease (COPD) or asthma, heart conditions (i.e., heart attack, heart disease, or other heart condition), pulmonary arterial hypertension, high blood pressure, diabetes or pre-diabetes, HIV, non-alcoholic fatty liver disease, or the hepatitis C virus	yes (345) no (904)
Wealth (latent)	Annual household income	<$20,000 annual household income (92) $20,000–$49,999 (253) $50,000–$84,999 (302) $85,000–$149,999 (336) ≥$150,000 (284)
	Educational attainment	<high school (96) high school (375) some college (332) Bachelor’s degree or higher (464)
	Home ownership	yes (969) rented for cash or occupied without payment of cash rent (298)
Civic Engagement (latent)	Self-reported likely voter	unlikely = score of 1–6 (218) likely = score of 7–10 (1037)
	Self-reported personal efficacy to solve problems in community	no difference (322) a little difference (527) some difference (334) a great deal of difference (70)
	Self-reported collective efficacy to solve problems in community	no difference (90) a little difference (271) some difference (573) a great deal of difference (323)
	Self-reported informal collaboration to solve a community problem	within the past 12 months (171) yes but not within the last 12 months (241) no (846)

## Results

We assessed demographic (Table 2), wealth (Table 3), civic engagement (Table 4), and health measures (Table 5) in our nationally representative sample. Our weighted sample demographics largely mirrored the national distribution of all adults: 51.6% female, 71.4% aged 25–64 years, 63.1% non-Hispanic White, and 53.2% married. The frequencies and percentages for our demographic measures were comparable to those from the Current Population Survey in all categories (Table 2).

**Table 2:** Demographic characteristics: Tufts Equity Survey, 2020 (*n* = 1267) vs. Current Population Survey, 2020

Factor	Equity Survey Totals n (weighted percentage)	2020 CPS March Supplement
**Gender**		
Female	631 (51.6%)	51.5%
Male	636 (48.4%)	48.5%
**Age (years)**		
18–24	55 (8.4%)	11.5%
25–34	165 (20.5%)	18.0%
35–44	187 (17.1%)	16.4%
45–54	188 (13.7%)	15.7%
55–64	317 (20.1%)	16.7%
65–74	236 (13.5%)	12.8%
≥ 75	119 (6.8%)	8.9%
**Race/Ethnicity**		
Hispanic	151 (16.4%)	16.7%
NH Black	117 (11.8%)	11.9%
NH White	893 (63.1%)	62.8%
Multiracial or other	106 (8.6%)	8.6%
**Marital Status**		
Married	769 (53.2%)	52.9%
Widowed	73 (4.8%)	5.8%
Divorced	134 (9.5%)	10.0%
Separated	18 (1.6%)	1.8%
Never married	210 (24.3%)	29.5%
Living with partner	63 (6.6%)	–

*Notes.* Source: Authors’ analysis of Tufts Equity Survey. Sample sizes are unweighted. Percentages have sample weights applied to be representative of U.S. population

*CPS* Current Population Survey, 2020 (U.S. Census Bureau), *NH* Non-Hispanic

**Table 3:** Wealth and socioeconomic characteristics: Tufts Equity Survey, 2020 (*n* = 1267) vs. Current Population Survey, 2020

Factor	Equity Survey Totals n (weighted percentage)	2020 CPS March Supplement
**Income**		
< $10,000	35 (3.6%)	3.5%
$10,000 to $14,999	31 (2.9%)	2.5%
$15,000 to $24,999	68 (7.1%)	5.8%
$25,000 to $34,999	88 (8.2%)	6.9%
$35,000 to $49,999	123 (10.0%)	10.4%
$50,000 to $74,999	211 (17.2%)	16.3%
$75,000 to $99,999	163 (13.7%)	13.3%
$100,000 to $149,999	264 (17.7%)	18.2%
$150,000 to $199,999	165 (11.7%)	10.3%
≥ $200,000	119 (7.9%)	12.8%
**Employment Status**		
Working	804 (65.2%)	60.2%
Laid off/looking for work	47 (5.6%)	3.2%
Retired/disabled	356 (22.7%)	24.4%
Not working (other)	60 (6.5%)	12.2%
**Education**		
Less than high school	96 (10.6%)	9.8%
High school	375 (28.3%)	27.8%
Some college	332 (27.8%)	27.6%
Bachelor’s degree or higher	464 (33.3%)	34.8%
**Home Ownership**		
Owned or being bought by participant or someone in their household	969 (70.9%)	70.1%
Rented for cash/occupied without payment of cash rent	298 (29.2%)	29.9%

*Notes.* Source: Authors’ analysis of Tufts Equity Survey, 2020. Sample sizes are unweighted. Percentages have sample weights applied to be representative of U.S. population

*CPS* Current Population Survey, 2020 (U.S. Census Bureau)

**Table 4:** Civic engagement measures in the U.S.: Tufts Equity Survey, 2020 (*n* = 1267)

Factor	Equity Survey Totals n (weighted percentage)
**Party Affiliation**	
Democrat	424 (35.4%)
Republican	398 (28.3%)
Other	445 (36.3%)
**Civic Engagement**	
None	776 (63.3%)
At least one	491 (36.7%)
**Ever worked together informally with someone or some group to solve a problem in the community (community problem-solving)**	
Yes, within the past 12 months	171 (13.4%)
Yes, but not within the past 12 months	241 (17.2%)
No	846 (68.5%)
Refused	9 (0.9%)
**Civic engagement: personal efficacy**	
Refused	14 (1.1%)
No difference at all	322 (24.7%)
A little difference	527 (41.3%)
Some difference	334 (26.9%)
A great deal of difference	70 (6.0%)
**Civic engagement: collective efficacy**	
Refused	10 (0.8%)
No difference at all	90 (7.5%)
A little difference	271 (20.9%)
Some difference	573 (44.7%)
A great deal of difference	323 (26.0%)
**Civic engagement: Regular local voting**	
Missing (not asked/refused/missing)	132 (13.6%)
Never vote in local elections	112 (10.9%)
Rarely vote in local elections	132 (12.1%)
Sometimes vote in local elections	276 (21.0%)
Always vote in local elections	615 (42.4%)
**Civic engagement: Plan to vote in 2020** (1 represents someone who definitely will not vote and 10 represents someone who definitely will vote)	
Refused	12 (1.3%)
1	112 (10.1%)
2–5	15 (10.4%)
6–9	133 (12.6%)
10	913 (65.7%)

*Source:* Authors’ analysis of Tufts Equity Survey, 2020. Sample sizes are unweighted. Percentages have sample weights applied to be representative of U.S. population

**Table 5:** Health outcomes and behaviors in the U.S.: Tufts Equity Survey, 2020 (*n* = 1267)

Factor	Equity Survey Totals n (weighted percentage)
**Self-Rated General Health**	
Refused	11 (1.0%)
Excellent	148 (12.3%)
Very good	487 (37.3%)
Good	426 (33.2%)
Fair	157 (12.6%)
Poor	38 (3.7%)
**Infectious Disease**	
Told by healthcare provider have COVID-19	8 (0.8%)
Has human immunodeficiency virus (HIV)	6 (0.4%)
Has hepatitis C virus (HCV)	10 (0.8%)
**Chronic Health Conditions**	
Pulmonary arterial hypertension	4 (0.3%)
High blood pressure	377 (24.1%)
Heart attack, heart disease, or other heart condition	92 (6.2%)
Cancer	82 (4.6%)
Chronic kidney disease	24 (1.5%)
Diabetes or pre-diabetes	176 (11.8%)
Asthma, chronic bronchitis, or chronic obstructive pulmonary disease (COPD)	138 (11.0%)
Mean body mass index (standard error)^a^	28.3 (0.21)
Obesity (body mass index ≥30 kg/m^2^)	406 (31.9%)
**Mental Health**	
Anxiety	118 (11.1%)
Depression	166 (14.2%)
Schizophrenia	3 (0.3%)
Mood disorder	11 (1.2%)
**Health Behaviors**	
Smoked ≥100 cigarettes in lifetime	478 (34.5%)
Ever vaped	185 (17.1%)
Alcohol use disorder	33 (2.6%)
Substance use disorder	28 (2.2%)
Opioid use disorder	11 (1.2%)
Has health insurance	1101 (84.2%)

*Source:* Authors’ analysis of Tufts Equity Survey, 2020. Sample sizes are unweighted. Percentages have sample weights applied to be representative of U.S. population

^a^Mean and standard error are both weighted values

Similarly, the wealth and socioeconomic characteristics in our weighted sample largely mirrored the national distributions from the Current Population Survey: 49.0% earned less than $75 K, 65.2% employed, 56.1% completed high school or some college while 33.3% completed a Bachelor’s degree or higher, and 70.9% owned their home (Table 3).

We found that nearly two-thirds of our sample reported not being civically engaged (63.3%) in activities such activism or community organizing. Approximately one-third of respondents (32.9%) reported some or a great deal of personal efficacy, while 70.7% reported some or a great deal of collective efficacy, and 65.7% definitely planned to vote in 2020. In terms of political affiliations, about one-third belonged to either the Democratic (35.4%) or Republican (28.3%) party. Nearly 7 in 10 (68.5%) reported that they had never worked together informally with another person or group to solve a problem in the community.

Approximately half (49.6%) of our respondents reported excellent or very good general health (Table 5). Nearly one-third of participants reported being obese (31.9%) and one-quarter of respondents reported having been diagnosed by a medical professional with high blood pressure (24.1%). Approximately one in seven participants reported having been diagnosed with depression (14.2%). Approximately one in 10 participants reported having been diagnosed with anxiety (11.1%), diabetes or pre-diabetes (11.8%), and asthma, chronic bronchitis, or chronic obstructive pulmonary disease (11.0%), while 6.2% reported having been diagnosed with a heart attack, heart disease, or other heart condition, and 4.6% reported a cancer diagnosis. In assessing health behaviors, we found that one-third of respondents reported ever smoking (34.5%) and 17.1% had ever vaped. The vast majority (84.2%) reported having private or public health insurance.

We report evidence of both major and everyday discrimination experiences in Supplemental Table 1. In general, the reported frequencies of everyday discrimination experiences were higher than those of major discrimination experiences.

### Relationships among health, wealth, and civic engagement constructs

To empirically test the hypothesized relationships among health, wealth, and civic engagement constructs shown in our conceptual framework (Fig. 1), we generated the illustrative model results shown in Fig. 2. The overall model structure that we hypothesized in Fig. 1 was supported in this illustrative example. For example, the covariance between the wealth and civic engagement indicators was significant (*p* = 0.013). Additionally, the wealth latent construct significantly predicted the general health indicator (β = 0.19; 95% CI = 0.01, 0.37; *p* = 0.035). The civic engagement latent construct was constrained at 1 so the significance of the relationship between civic engagement and health cannot be determined in the main model; however, in a second version of the model where the wealth latent construct was constrained at 1, the civic engagement construct was a significant predictor of the health variable (β = 0.44; 95% CI = 0.11, 0.77; *p* = 0.010).

In the full structural equation model (Supplemental Table 2), we observed moderately strong positive associations between the wealth construct and each of the indicators for civic engagement (coefficients between 0.38 and 0.47). The wealth indicators were each significantly associated with the latent construct for wealth (income was constrained to 1, β = 0.40 for education with *p* = 0.026, and β = 0.34 for home ownership with *p* = 0.001). Similarly, the civic engagement indicators were each significantly associated with the latent construct of civic engagement (*p* < 0.01 for each indicator). Additionally, the direction of each health, wealth, and civic engagement indicator was consistent with the hypothesized relationships, suggesting that our constructs were valid. For example, we observed that smoking was associated with a 46% lower likelihood of reporting excellent or very good health (*p* < 0.001), having been diagnosed with at least one chronic health condition was associated with a 39% lower likelihood of reporting excellent or very good health (*p* = 0.005), and each point increase in BMI was associated with a 9% lower likelihood of reporting excellent or very good health (*p* < 0.001). The effect estimates for having been diagnosed with depression or anxiety and having a substance use disorder were negative in relation to self-reported general health, as expected, but were not significant (*p* = 0.056 and *p* = 0.359, respectively). For comparison, the results of the structural equation model were generally consistent with bivariate associations between each of the indicator variables and the observed overall physical health variable (Supplemental Table 3). At least one level of each wealth and civic engagement variable was significantly associated with the overall physical health variable (*p* < 0.05).

## Discussion

The goal of the current study was to help fill a gap in the extant literature by assessing holistic connections across all three equity domains—health, wealth, and civic engagement. Whereas current literature has explored inequities in health and wealth, in health and civic engagement, and in civic engagement and wealth, to our knowledge, no prior research has explored the relationships among these three domains. We developed a conceptual framework and conducted a cross-sectional, online survey between May 29–June 20, 2020 designed to be representative of the adult U.S. population. Through our statistical models we explored the complex web of domains that contribute to and hinder equity.

We first noted that the demographic, wealth, health, and civic engagement characteristics of our sample were comparable to those of the U.S. population, as our frequencies and distributions for similar measures were in line with those from the U.S. Census Bureau [44]. We found that many forms of civic engagement were relatively uncommon, and that poor health outcomes were common, as also noted in the Current Population Survey. In our structural equation model illustrating one set of possible interrelationships among wealth, civic engagement, and health, we found evidence that all three domains together are associated with self-reported general physical health. We suggest that a complex web of factors across each of the health, wealth, and civic engagement domains affects individual and community health and wellbeing.

Traditional research has focused on singular or dual dimensions of equity, such as wealth or wealth and health [1, 3], noting that inequalities in wealth are associated with inequalities in health [4–6], or health and civic engagement where, for instance, increased well-being, physical health and mental health are associated with increased civic engagement [8], and that poor health earlier in life is associated with lower levels of civic engagement during the latter years of life [9, 10], thus examining equity through a narrow lens. To understand the complex drivers of equity, research must look at the connections among three important areas of equity: health, wealth, and civic engagement. We suggested one approach to doing so with our structural equation model. We found significant covariance between our wealth and civic engagement constructs. Associations between our wealth and health constructs were also significant, tying together our tri-focal equity domains, and they were consistent with recent research, indicating that associations among these domains may be bidirectional. As noted by Knapp and Wong, for instance, adverse mental health harms individuals’ economic circumstances, and poverty contributes to adverse mental health [7].

Our findings are consistent with those in the literature relating two domains at a time. For example, we observed significant associations between indicators of wealth and health in the SEM and in the bivariate regression analyses. Similarly, others have found that adults who have lower levels of income are almost five times as likely to report being in fair or poor health as adults with family incomes at or above 400% of the federal poverty level (Braveman & Egerter, 2008). When looking at life expectancy, the gap between rich and poor Americans has been widening since the 1970s [5] with the wealthiest 1% living 10–15 years longer than the poorest 1% of the population [4].

In support of our observations that health and civic engagement constructs were associated, and considerable previous research suggests that bidirectional relationships may exist. Engaging in various forms of civic life including voting, activism, community organizing, and direct community service provides an avenue for social change and is associated with positive health outcomes [45]. And conversely, prior studies suggest that increased well-being, physical health and mental health are associated with increased civic engagement [8]. While the ability to participate in civic life and its salutary effects on health may vary among people with different chronic conditions [46, 47], positive associations hold across different types of engagement (e.g., voting, volunteering) [8]. Understanding health status is crucial to understanding who participates in civic action. For example, the few longitudinal analyses that have been conducted on this topic suggest that poor health earlier in life is associated with lower levels of civic engagement later in life [9, 10].

Finally, we observed significant correlations between measures of wealth and civic engagement suggesting that a full understanding of the drivers for equity needs to consider both of these domains. Others have observed that these associations have persisted, and increased, over time. Declining trends in volunteering and associational membership in the United States, for instance, are associated with higher rates of income inequality (Costa & Kahn, 2003 [12]), and Lim [48] suggests that economic inequality negatively impacts civic engagement. In addition, other forms of social capital, such as trust, are strongly and negatively affected by income inequality, which in turn lowers rates of community participation [14, 15]. Further, considering the wealth and civic engagement domains, and consistent with our findings, researchers have consistently found associations between wealth and social class, and civic engagement behaviors [11]. Americans impacted by income inequality are also less likely to participate in other forms of community engagement such as recreational, civic, and educational groups [13].

### Limitations

Our findings should be considered in light of several limitations. The cross-sectional nature of our survey precludes assessment of causal associations. The SEM provided one illustrative example of how indicators of health, wealth, and civic engagement may relate, but should not be construed as the only or the most comprehensive way to capture the complex interrelationships among the three domains. Additionally, while racial/ethnic groups were sampled in proportion to their national distribution, some groups (Blacks and Latinos) had a lower survey completion rate, and thus are slightly underrepresented in the unweighted data. However, our data are weighted back to population dynamics in the U.S. Given the nature of the KnowledgePanel data, some core measures were collected prior to our equity-based survey, and some explanatory measures may have changed during the timeframe between the administration of the Ipsos profile surveys and the Tufts equity survey. In addition, not all of our measures were standardized or validated measures. Sampling bias is also possible for some of our measures as our sample was weighted on typical demographic measures but not on behavioral measures (e.g., smoking).

Despite these limitations, our study has several strengths. We employed a probability-based weighting approach, which further enhanced the external validity of our results. Ipsos’ Knowledge Panel recruitment methodology uses the same or similar quality standards as mandated by the Office of Management and Budget in the “List of Standards for Statistical Surveys,” which indicates that “Agencies must develop a survey design, including…selecting samples using generally accepted statistical methods (e.g., probabilistic methods that can provide estimates of sampling error)” [49]. Our survey was timely during the pandemic and the concurrent Black Lives Matter movement. Further, our proposed conceptual model is empirically supported by our data and analyses. We look forward to building upon this model to explore the multi-level and nuanced components of equity across the U.S. in the months and years to come as we add further waves of data collection. Additionally, a de-identified version of these data will be made available upon request to researchers who wish to explore different components of equity and associations that we have yet to explore. Exploration of some of the salient measures collected in our survey, as well as bivariate comparisons and visualizations of results are available at our public facing website (https://equityresearch.tufts.edu/).

## Conclusion

Through analyses of data from our cross-sectional survey designed to be representative of the U.S. population in 2020, we explored holistic connections across the domains of equity in health, wealth, and civic engagement. In our structural equation and regression models, we identified three-way relationships between these domains, consistent with the hypothesized relationships in our conceptual model. Given empirical associations among measures of health, wealth and civic engagement, future research should holistically consider assessing all of these domains, using additional sophisticated approaches, in order to generate a more comprehensive understanding of health equity. Understanding the mechanisms that tie health, wealth, and civic engagement together would have important implications for the development of policies and programs aimed at achieving equity in communities across the globe. It will also help us shift the paradigm from documenting inequity to identifying targets for change.

## Supplementary Information

**Additional file 1: Supplemental Table 1.** Discrimination experiences in the U.S., 2020 (*n* = 1267).

**Additional file 2: Supplemental Table 2.** Structural equation model output.

**Additional file 3: Supplemental Table 3.** Bivariate logistic regression associations between structural equation model variables and self-reported physical health.

## Data Availability

A de-identified version of these data will be made available upon request to researchers who wish to explore different components of equity and associations that we have yet to explore. Exploration of some of the salient measures collected in our survey, as well as bivariate comparisons and visualizations of results, are available at our public facing website (https://equityresearch.tufts.edu/).
