# Supplementary material for: Assessing equity in health, wealth, and civic engagement: a nationally representative survey, United States, 2020
Source: Int J Equity Health. 2022 Jan 28;21:12. doi: 10.1186/s12939-021-01609-w (PMC8795944; doi:10.1186/s12939-021-01609-w)
Supplement: Supplementary file 1 — Additional file 1: Supplemental Table 1. Discrimination experiences in the U.S., 2020 (n = 1267). [file 12939_2021_1609_MOESM1_ESM.docx]

**Supplemental Table 1.** Discrimination experiences in the U.S., 2020 (n=1,267)

| **Measures** | **Equity Survey Totals (weighted percentage)** |
| --- | --- |
| **Major Discrimination Experiences** |  |
| You have been unfairly stopped, searched, questioned, physically threatened or abused by the police |  |
| Refused | 15 (1.3%) |
| Never | 1037 (79.9%) |
| Once or twice | 98 (8.5%) |
| Three or more times | 23 (2.4%) |
| Not in the past year but in the past | 94 (7.9%) |
| Someone you know has been unfairly stopped, searched, questioned, physically threatened or abused by the police |  |
| Refused | 17 (1.5%) |
| Never | 839 (63.8%) |
| Once or twice | 186 (15.6%) |
| Three or more times | 72 (6.7%) |
| Not in the past year but in the past | 153 (12.5%) |
| You were mistaken for someone else of your same race/ethnicity (who may not look like you at all) |  |
| Refused | 17 (1.4%) |
| Never | 946 (72.3%) |
| Once or twice | 160 (14.2%) |
| Three or more times | 49 (4.3%) |
| Not in the past year but in the past | 95 (7.8%) |
| You have been unfairly prevented from having access to a service or been treated unfairly by a service provider |  |
| Refused | 17 (1.5%) |
| Never | 975 (75.1%) |
| Once or twice | 142 (11.6%) |
| Three or more times | 35 (3.3%) |
| Not in the past year but in the past | 98 (8.5%) |
| **Everyday Discrimination Experiences** |  |
| Being treated with less courtesy or respect than other people |  |
| Refused | 15 (1.3%) |
| Never | 531 (40.0%) |
| Rarely | 477 (36.9%) |
| Sometimes | 225 (19.9%) |
| Frequently | 19 (1.8%) |
| Feeling that people act as if they are afraid of you |  |
| Refused | 17 (1.4%) |
| Never | 897 (69.5%) |
| Rarely | 234 (19.0%) |
| Sometimes | 107 (9.3%) |
| Frequently | 12 (0.8%) |
| Receiving poorer service than others in restaurants and stores |  |
| Refused | 18 (1.7%) |
| Never | 668 (52.1%) |
| Rarely | 406 (30.7%) |
| Sometimes | 160 (13.9%) |
| Frequently | 15 (1.5%) |
