# Supplementary material for: Assessing equity in health, wealth, and civic engagement: a nationally representative survey, United States, 2020
Source: Int J Equity Health. 2022 Jan 28;21:12. doi: 10.1186/s12939-021-01609-w (PMC8795944; doi:10.1186/s12939-021-01609-w)
Supplement: Supplementary file 2 — Additional file 2: Supplemental Table 2. Structural equation model output. [file 12939_2021_1609_MOESM2_ESM.docx]

**Supplemental Table 2.** Structural equation model output

| **Predicted**  **(variable or construct)** | **Indicator**  **(variable, construct, or level)** | **coefficient** | **95% C.I.** | **p** |
| --- | --- | --- | --- | --- |
| Health (measured outcome) | civic engagement construct | constrained | | |
|  | wealth construct | 0.19 | (0.01, 0.37) | 0.035 |
|  | body mass index | -0.10 | (-0.13, -0.07) | <0.001 |
|  | smoking | -0.63 | (-0.93, -0.32) | <0.001 |
|  | depression and/or anxiety | -0.38 | (-0.77, 0.01) | 0.056 |
|  | substance use disorder | -0.24 | (-0.75, 0.27) | 0.359 |
|  | chronic health condition(s) | -0.49 | (-0.83, -0.15) | 0.005 |
|  | constant | 3.30 | (2.49, 4.12) | <0.001 |
| Likely voter | civic engagement construct | 2.28 | (0.59, 3.98) | 0.008 |
|  | constant | 1.41 | (1.22, 1.60) | <0.001 |
| Personal efficacy | civic engagement construct | 10.64 | (3.64, 17.64) | 0.003 |
| Collective efficacy | civic engagement construct | 10.49 | (3.29, 17.68) | 0.004 |
| Community problem solving | civic engagement construct | 4.73 | (1.59, 7.87) | 0.003 |
| Income | wealth construct | constrained | | |
| Educational attainment | wealth construct | 0.40 | (0.05, 0.74) | 0.026 |
| Home ownership | wealth construct | 0.34 | (0.13, 0.55) | 0.001 |
|  | constant | 1.03 | (0.86, 1.20) | <0.001 |
| Personal efficacy  (reference: no difference) | a little difference | -2.35 | (-2.80, -1.89) |  |
|  | some difference | 1.54 | (1.16, 1.92) |  |
|  | a great deal of difference | 5.41 | (4.44, 6.38) |  |
| Collective efficacy  (reference: no difference) | a little difference | -5.08 | (-5.89, -4.28) |  |
|  | some difference | -1.87 | (-2.26, -1.49) |  |
|  | a great deal of difference | 2.23 | (1.80, 2.67) |  |
| Community problem solving (reference: no difference) | yes, but not within the past 12 months | 1.06 | (0.88, 1.25) |  |
|  | yes, within the past 12 months | 2.38 | (2.13, 2.64) |  |
| Income (reference: <$20,000) | $20,000-$49,000 | -4.08 | (-5.72, -2.45) |  |
|  | $50,000-$84,999 | -1.47 | (-2.12, -0.82) |  |
|  | $85,000-$149,999 | 0.53 | (0.20, 0.85) |  |
|  | ≥$150,000 | 2.65 | (1.54, 3.76) |  |
| Educational attainment (reference: less than high school) | high school | -2.50 | (-2.81, -2.19) |  |
|  | some college | -0.57 | (-0.73, -0.41) |  |
|  | Bachelor’s or higher | 0.82 | (0.65, 0.99) |  |
| Variance (civic engagement construct) |  | 0.08 | (0.02, 0.30) |  |
| Variance (wealth construct) |  | 6.53 | (2.01, 21.26) |  |
| Covariance (civic engagement and wealth constructs) |  | 0.18 | (0.04, 0.33) | 0.013 |
