# Supplementary material for: Assessing equity in health, wealth, and civic engagement: a nationally representative survey, United States, 2020
Source: Int J Equity Health. 2022 Jan 28;21:12. doi: 10.1186/s12939-021-01609-w (PMC8795944; doi:10.1186/s12939-021-01609-w)
Supplement: Supplementary file 3 — Additional file 3: Supplemental Table 3. Bivariate logistic regression associations between structural equation model variables and self-reported physical health. [file 12939_2021_1609_MOESM3_ESM.docx]

**Supplemental Table 3.** Bivariate logistic regression associations between structural equation model variables and self-reported physical health

| **Variable** | **Odds ratio (95% confidence interval)** |
| --- | --- |
| Body mass index (continuous) | 0.90 (0.88, 0.92) |
| Ever smoked >100 cigarettes (dichotomous) | 0.46 (0.36, 0.59) |
| Self-reported diagnosis of depression or anxiety (dichotomous) | 0.47 (0.33, 0.67) |
| Self-reported being told by a healthcare professional that they had any of: a substance or alcohol or opioid-use disorder or self-reported using prescription pain medication not prescribed to them by a doctor (dichotomous) | 0.77 (0.48, 1.21) |
| Self-reported diagnosis by a healthcare professional of any of: chronic kidney disease, chronic obstructive pulmonary disease (COPD) or asthma, heart conditions (i.e., heart attack, heart disease, or other heart condition), pulmonary arterial hypertension, high blood pressure, diabetes or pre-diabetes, HIV, non-alcoholic fatty liver disease, or the hepatitis C virus (dichotomous) | 0.48 (0.36, 0.64) |
| Annual household income (referent: <$20,000) |  |
| $20,000-$49,000 | 1.53 (0.87, 2.71) |
| $50,000-$84,999 | 2.80 (1.61, 4.86) |
| $85,000-$149,999 | 3.25 (1.88, 5.62) |
| ≥$150,000 | 4.98 (2.85, 8.71) |
| Educational attainment (referent: < than high school) |  |
| High school | 1.34 (0.80, 2.23) |
| Some college | 1.98 (1.18, 3.33) |
| Bachelor’s or higher | 3.55 (2.14, 5.89) |
| Home ownership (dichotomous; referent: rented for cash or occupied without payment of cash rent) | 1.57 (1.17, 2.10) |
| Self-reported likely voter (dichotomous; referent: self-reported score of 1-6) | 1.78 (1.28, 2.47) |
| Self-reported personal efficacy to solve problems in the community (referent: no difference) |  |
| A little difference | 1.61 (1.18, 2.21) |
| Some difference | 2.65 (1.86, 3.78) |
| A great deal of difference | 2.38 (1.29, 4.39) |
| Self-reported collective efficacy to solve problems in the community (referent: no difference) |  |
| A little difference | 1.55 (0.88, 2.73) |
| Some difference | 2.47 (1.45, 4.21) |
| A great deal of difference | 3.34 (1.92, 5.84) |
| Self-reported informal collaboration to solve a community problem (referent: no) |  |
| Yes, but not within the past 12 months | 0.96 (0.69, 1.32) |
| Yes, within the last 12 months | 2.67 (1.76, 4.05) |

*Note.* Self-reported physical health responses were collapsed into “very good/excellent” compared to “poor/fair/good.”
